# Study of the Characteristics of Ba_0.6_Sr_0.4_Ti_1-x_Mn_x_O_3_-Film Resistance Random Access Memory Devices

**DOI:** 10.3390/mi15091143

**Published:** 2024-09-12

**Authors:** Kai-Huang Chen, Chien-Min Cheng, Ming-Cheng Kao, Yun-Han Kao, Shen-Feng Lin

**Affiliations:** 1 Department of Electronic Engineering, Center for Environmental Toxin and Emerging-Contaminant Research, Super Micro Mass Research & Technology Center, Cheng Shiu University, Kaohsiung 83347, Taiwan; 5977@gcloud.csu.edu.tw (K.-H.C.);; 2 Department of Electronic Engineering, Southern Taiwan University of Science and Technology, Tainan 710301, Taiwan; 3 Department of Information and Communication Engineering, Chaoyang University of Technology, Taichung 413310, Taiwan; kmc@cyut.edu.tw

**Keywords:** conduction mechanism, BaSrTiMnO_3_, resistance random access memory, electronic hopping distance

## Abstract

In this study, Ba_0.6_Sr_0.4_Ti_1-x_Mn_x_O_3_ ceramics were fabricated by a novel ball milling technique followed by spin-coating to produce thin-film resistive memories. Measurements were made using field emission scanning electron microscopes, atomic force microscopes, X-ray diffractometers, and precision power meters to observe, analyze, and calculate surface microstructures, roughness, crystalline phases, half-height widths, and memory characteristics. Firstly, the effect of different sintering methods with different substitution ratios of Mn^4+^ for Ti^4+^ was studied. The surface microstructural changes of the films prepared by the one-time sintering method were compared with those of the solid-state reaction method, and the effects of substituting a small amount of Ti^4+^ with Mn^4+^ on the physical properties were analyzed. Finally, the optimal parameters obtained in the first part of the experiment were used for the fabrication of the thin-film resistive memory devices. The voltage and current characteristics, continuous operation times, conduction mechanisms, activation energies, and hopping distances of two types of thin-film resistive memory devices, BST and BSTM, were measured and studied under different compliance currents.

## 1. Introduction

Since the invention of memory, many different types of memory have been developed, which can be divided into two main groups: volatile memory and non-volatile memory [1,2,3,4,5,6,7,8,9,10,11,12,13,14,15]. The difference between the two is that data stored in volatile memories such as dynamic random access memory (DRAM) and static random access memory (SRAM) disappear when externally applied power is removed. These two types of memory have simple structures, fast access speeds, and low power consumption, making them suitable for fast computation in coprocessors and the like [16]. In contrast, data stored in non-volatile memory (NVM) remain in the memory element when the externally applied power is removed or terminated. The next-generation NVMs developed to date include magneto resistive memory (MRAM), phase-change memory (PRAM), ferroelectric memory (FRAM), and resistive memory (RRAM) [17,18,19]. Among the above types of NVMs, resistive memory is one of the most promising types of memory, which has many advantages such as fast write and erase, simple component structure, low operating voltage, low power consumption, etc. Therefore, many researchers and scholars are actively involved in this topic to explore it more deeply and apply it to more products. In the past, the original *I-V* and *C-V* characteristics of ABO_3_ ferroelectric oxide materials were widely discussed and observed in non-volatile ferroelectric RAM devices. Recently, various ABO_3_ oxide materials such as BaSrTiO_3_ (BST) and SrBiTiO_3_ (SBT) films have started to be discussed and applied in resistive random access memory devices because of their ultra-high resistive switching current ratio and excellent memory window properties for Internet of things (IOT) applications in future. According to the literature, proper addition of Mn can effectively increase the switching speed, memory window, and number of operations, and reduce the electron density of the memory [20,21,22,23,24,25].

The new ball milling technology used is different from the general solid-state reaction method for one-time sintering of the materials in this study. In the sintering process of the traditional solid-state reaction method, crystal grains are initially grown during calcination. When the sintering is performed again, an insufficient or excessive reaction time results in the growth of the grains because of the influence of thermal energy in the subsequent film production process.

This experiment compares the differences between two sintering methods used to produce films in the new ball milling technology and investigates the effect of substituting Ti^4+^ with different ratios of Mn^4+^ on the physical properties. Then, the optimal parameters obtained in the first part of the experiment are used for the fabrication of the resistive memory element, and the voltage and current characteristics, continuous operation times, conduction mechanisms, activation energies, and hopping distances of two types of thin-film resistive memories, BST and BSTM, are measured and studied under different compliance currents.

## 2. Experimental Details

**Figure 1:**
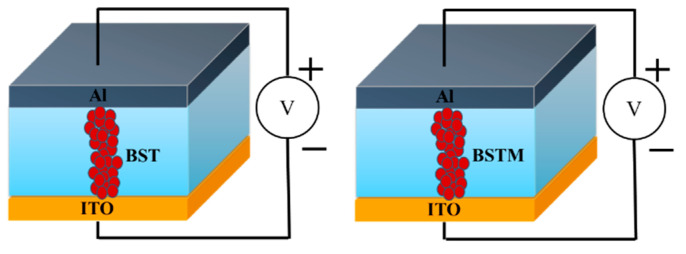
Structure diagram of BST- and BSTM-film RRAM devices.

The experiment was divided into three stages. In the first stage, the indium tin oxide (ITO) transparent conductive glass substrate was cleaned and two different sintering methods, the one-time sintering method and the solid-state reaction method, were used to prepare BSTM and BST precursor solutions, which were deposited on the ITO glass substrate at an initial speed of 1500 rpm/10 s and a final speed of 5000 rpm/30 s, in order to compare the quality of the films produced by the two methods. In the second stage, different ratios for Ba_0.6_Sr_0.4_Ti_1-x_Mn_x_O_3_ (x = 0.05, 0.1, 0.15, 0.2) were investigated and compared with Ba_0.6_Sr_0.4_TiO_3_ films by FE-SEM (field emission–scanning electron microscopy, Regulus8100, KEYENCE, Taipei, Taiwan), X-ray diffractometer (XRD, BTX III, Bruker, Billerica, MA, USA), and other instruments. For the metal–insulator–metal (MIM) structure fabricated, the *I-V* curve properties of the BSTM and BST film devices were obtained and observed. In the third stage, BSTM and BST memory devices were fabricated by thermal vapor deposition with aluminum (Al) top electrodes. Their structure is shown in Figure 1, and the differences in the characteristics of the memory devices made from the two films were investigated under different compliance currents by semiconductor parameter analyzer (Agilent B1500A, Taoyuan, Taiwan). Then, the electronic activation energies of the devices were analyzed and calculated from measurements at different temperatures.

## 3. Results and Discussion

**Figure 2:**
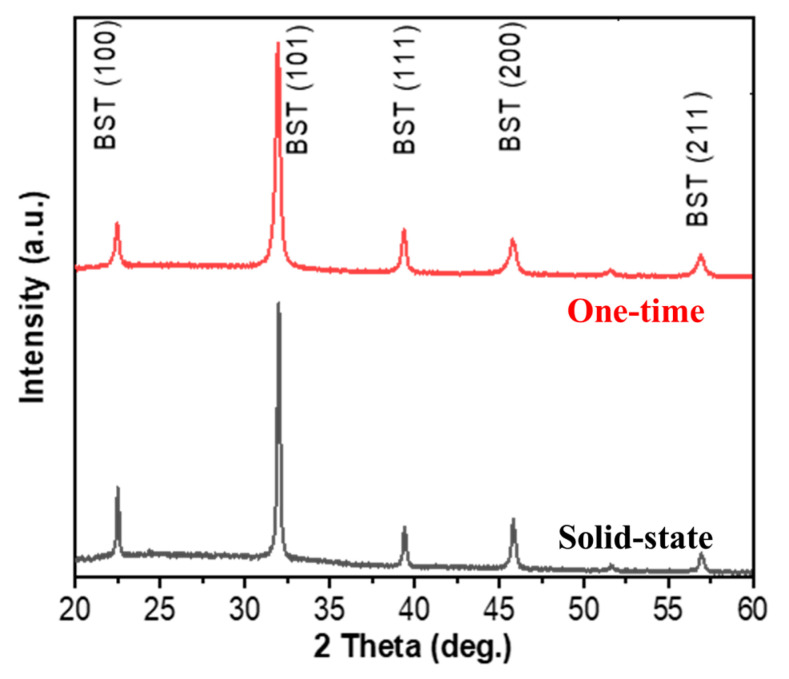
XRD patterns of the BST material for one-time sintering method and solid-state reaction method.

The XRD patterns of films prepared after sintering BST powder by the one-time sintering method and the solid-state reaction method at 1300 °C/4 h indicated that the crystalline phases of the BST films were (100), (101), (111), (200), and (211), as shown in Figure 2. The full width at half maximum (FWHM) of the two stronger peaks 101 and 111 were calculated for the solid-state reaction method and the one-time sintering method, respectively, and the theoretical densities and crystallinity were determined, as shown in Table 1.

A smaller peak FWHM indicates better crystallinity. FWHM(101) was 0.385 and 0.245 for the solid-state reaction method and the one-time sintering method, respectively. FWHM(111) was 0.324 and 0.235 for the solid-state reaction method and the one-time sintering method, respectively. After calculation, the crystallinity was found to be 96.123% for the solid-state reaction method and 97.223% for the one-time sintering method. Comparing the two, the crystallinity of the one-time sintering method is better. The grain size was then calculated by the Debye–Scherrer formula, as shown in Equation (1), where D is the nanoparticles’ crystalline size, K represents the Scherrer constant (0.98), λ denotes the wavelength (1.54), and β denotes the full width at half maximum (FWHM). In addition, the error of the measured parameters was about 3–5%.

**Figure 3:**
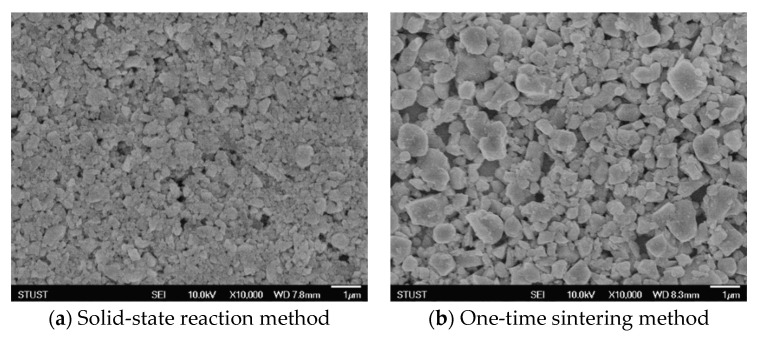
FE-SEM images of the BST material for one-time sintering method and solid-state reaction method.

The crystal size of the BST film was 0.174 μm for the solid-state reaction method and 0.268 μm for the one-time sintering method. A significant difference in the crystal size between the two methods can be seen in Figure 3. The use of solid-state reaction to produce films is effective in reducing grain size, but the increase in temperature during the sintering growth process causes the crystals to grow gradually. If the temperature is too high or the reaction time too long, the crystal grains will grow too fast and reassemble into new structures; on the other hand, at lower temperatures, the carriers will not be able to migrate to their correct positions due to insufficient thermal energy, resulting in a decrease in the degree of crystallinity. The one-time sintering method uses a single high-temperature sintering process to enable the carrier to obtain thermal energy to move to the desired position for structure formation, thereby avoiding the internal stress accumulated in the structure by calcination and sintering in the solid-state reaction method, and improving the quality of subsequent film production. On the basis of these experimental results, it was decided to use the one-time sintering method for the production of subsequent films.

(1) $$\tau =\frac{K\lambda }{\beta cos\;\theta }$$

The changes in surface roughness of the films after deposition by the two fabrication methods were investigated by atomic force microscope (AFM, Bruker Dimension ICON, USA). Table 2 shows that the average roughness, root mean square roughness, and maximum height roughness of the solid-state reaction method were 72.1 nm, 93.7 nm, and 909 nm, while those of the one-time sintering method were 125 nm, 159 nm, and 1264 nm, respectively. At the sintering temperature of 1300 °C, the solid-state reaction method resulted in a lower surface roughness than the one-time sintering method due to poor crystallinity.

**Figure 4:**
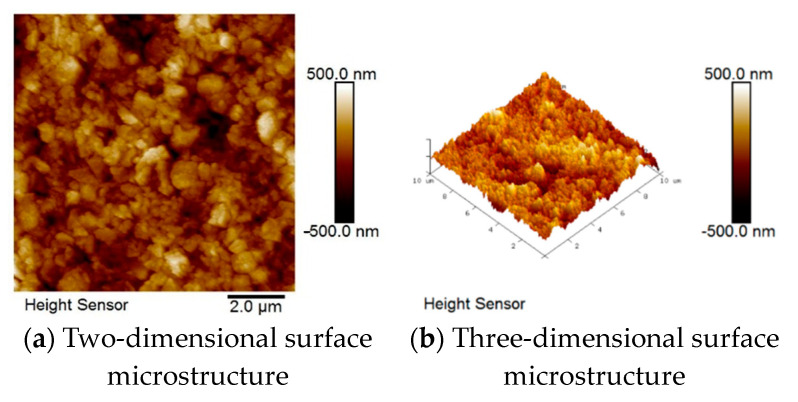
AFM diagram of BST materials for the solid-state reaction method.

**Figure 5:**
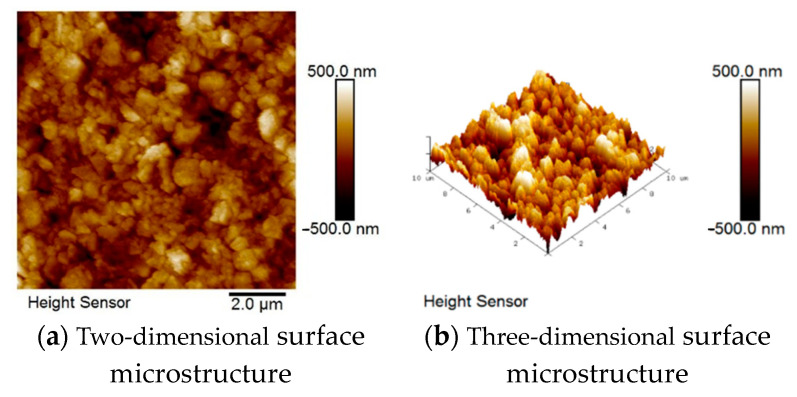
AFM diagram of BST materials for the one-time sintering method.

In Figure 4 and Figure 5, the dark-brown color indicates the low points in the sample; the lighter the color, the higher the point. It can be clearly seen that the color distribution in Figure 4a is more uniform than that in Figure 5a, confirming that the solid-state reaction method results in lower roughness as shown by the measured values. The 3D surface microstructures in Figure 4b and Figure 5b clearly show the grain growth and deposition on the surface of the specimen.

**Figure 6:**
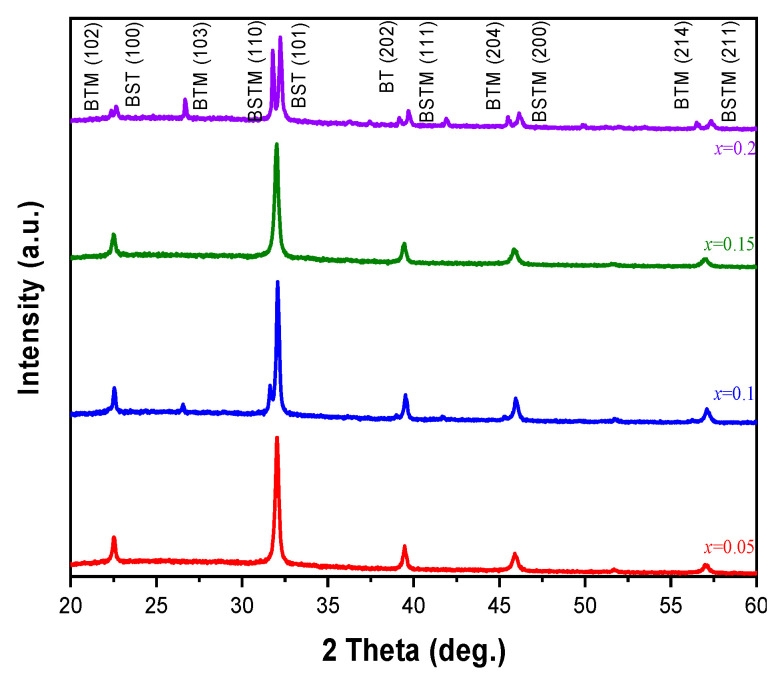
XRD patterns of BSTM materials for different Mn proportions.

Figure 6 shows the XRD patterns of films formed by coating the substrate with precursor solutions prepared from BSTM powders in different proportions by the one-time sintering method, and it shows that the crystalline phases of the BSTM films were (110), (111), (200), and (211), and the crystalline phases of the BST were (100) and (101). The full width at half maximum (FWHM) of the two stronger peaks 110 and 111 for BSTM films prepared at different proportions was calculated to determine the theoretical densities and crystallinity, as shown in Table 3.

**Figure 7:**
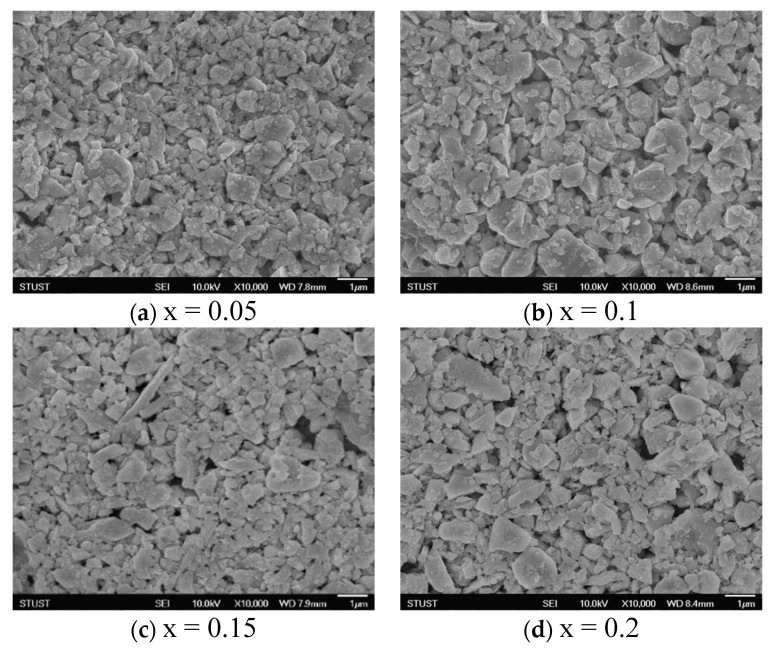
FE-SEM images for different Mn proportions of BSTM materials.

The addition of 0.1 mole of Mn resulted in 0.234 for FWHM (110) and 0.227 for FWHM (111), which were the lowest compared to other proportions. In addition, it had a maximum crystallinity of 98.577, a theoretical density of 5.5 g/cm^3^, and an average grain size of 0.277 μm. Therefore, it can be concluded that this proportion produced a better structural state and more perfect grain growth. When 0.15 and 0.2 mole of Mn are added, the over-addition of Mn leads to a decrease in crystallinity and collapse of the crystal structure. When 0.2 mole of Mn is added, because the radius of Mn^4+^ ions is 0.053 nm and the radius of Ti^4+^ ions is 0.0605 nm, the over-addition will cause the B site to shift its internal center, which will not be able to satisfy the valence bond required for the formation of the ABO_3_ structure during the crystallization process. As noted in the literature, the addition of Mn at x ≤ 0.35 produces Mn^2+^ with an ion radius of 0.067 nm, whereas the ion radii of Ba^2+^ and Sr^2+^ are 0.135 nm and 0.118 nm, respectively. Because Mn^2+^ and Mn^4+^ ions are small enough to enter the A-site in the crystal lattice, they cause displacement or recrystallization, resulting in the formation of impurity and recrystallized phases [9]. The experimental results show that the appropriate addition of Mn can effectively improve the grain growth and film quality, as in the surface microstructure state shown in Figure 7. The 0.1 mole Mn of BTM film exhibits excellent theoretical density (g/cm^3^), average grain diameter (μm), and crystallinity (%), as shown in Table 3.

**Figure 8:**
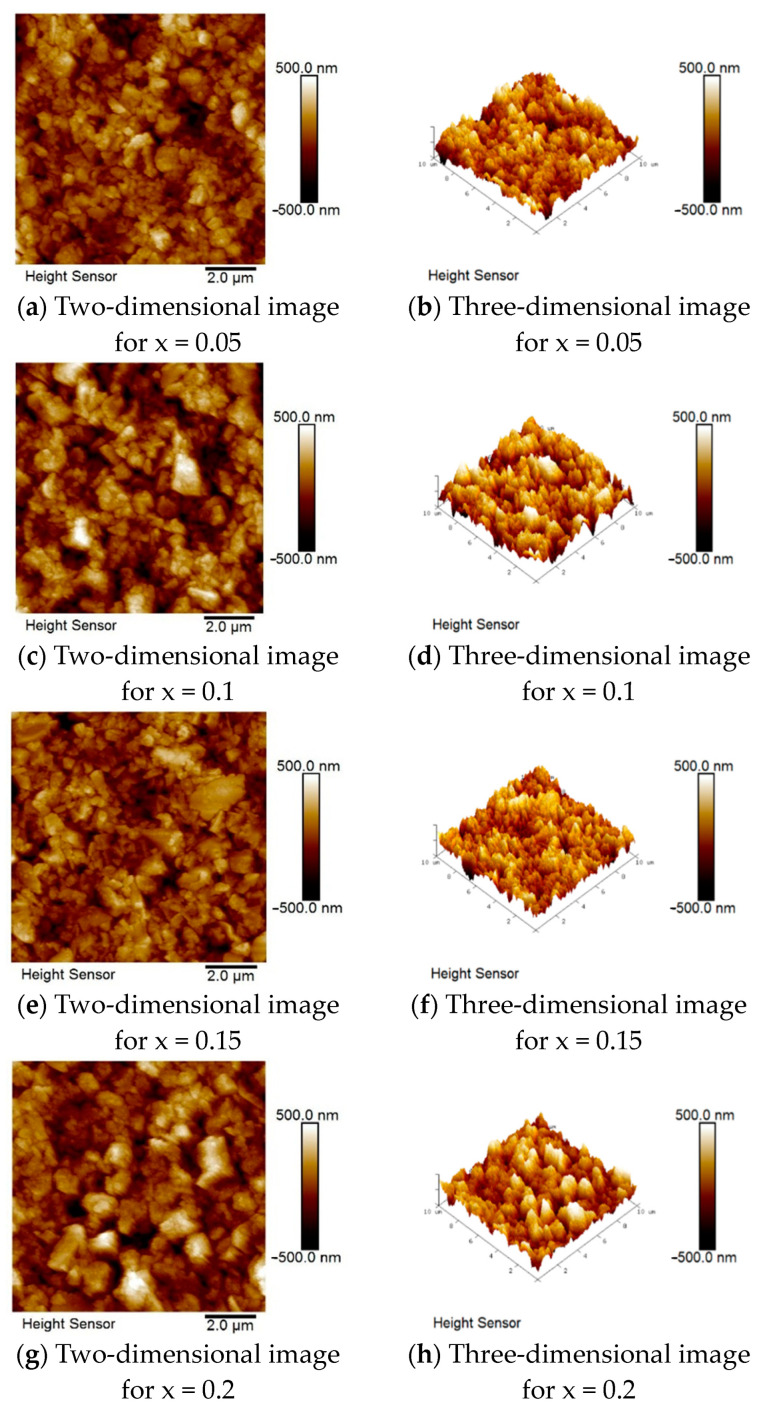
AFM surface images for different Mn proportions of BSTM materials.

Figure 8 shows the AFM images for each proportion, in which the surface deposition state of each film sample is observed from the 3D image of its surface microstructure. As shown in Table 4, the average roughness was 86.5 nm, the root mean square roughness was 111 nm, and the maximum height roughness was 1044 nm with the addition of 0.1 mole of Mn, which are slightly lower than those of other additive ratios. Therefore, it can be concluded that with the addition of 0.1 mole of Mn, the film surfaces are flatter and have fewer defects. In addition, the number of defects indirectly contributes to problems such as current leakage and energy barriers in subsequent device fabrication. The higher the manganese addition ratio, the greater the surface roughness, which may be due to structural collapse or secondary crystalline phases formed by recrystallization during the reaction process in the case of heavy doping.

**Figure 9:**
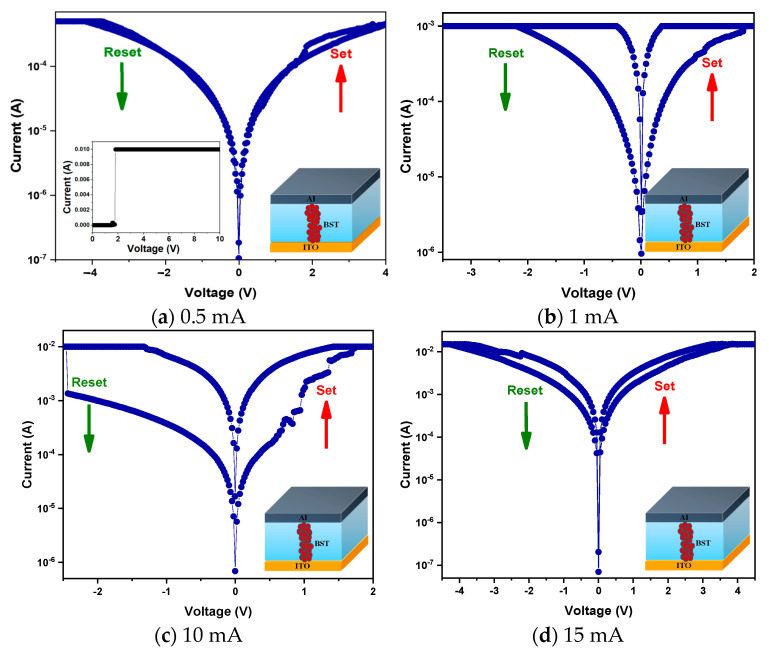
The *I-V* curves of BST-film RRAM devices for different compliance currents. (red symbol: oxygen ions).

Next, the memory window of the BST resistive memory was determined by scanning back and forth with different compliance currents, as shown in Figure 9. As seen in Figure 9a, for 0.5 mA compliance current, there was no clear transition state between high and low resistance states (abbreviated as HRS and LRS) because the formation and breakage of resistive filaments in the element was slow or had not yet occurred due to insufficient current. As shown in Figure 9b, for 1 mA compliance current, the memory window appeared but a cutoff current was generated in the LRS, due to insufficient current being supplied so that the device did not have a large enough operating current during the switching process. And as seen in Figure 9c, for 10 mA compliance current, the memory window increased significantly, with an on/off ratio of about 1 and a leakage current of about 10^−3^ A. But for 15 mA compliance current, the formation of excessive resistive filaments leads to the near collapse of the film due to the transient passage of too many carriers, resulting in a significant reduction of the memory window, and the excessive resistive filaments tend to prevent the complete breakage of the device during the switching process, resulting in an increase in leakage current, as shown in Figure 9d. Therefore, it can be concluded from the experiments that the most optimal characteristics of the BST memory element are obtained at 10 mA current.

**Figure 10:**
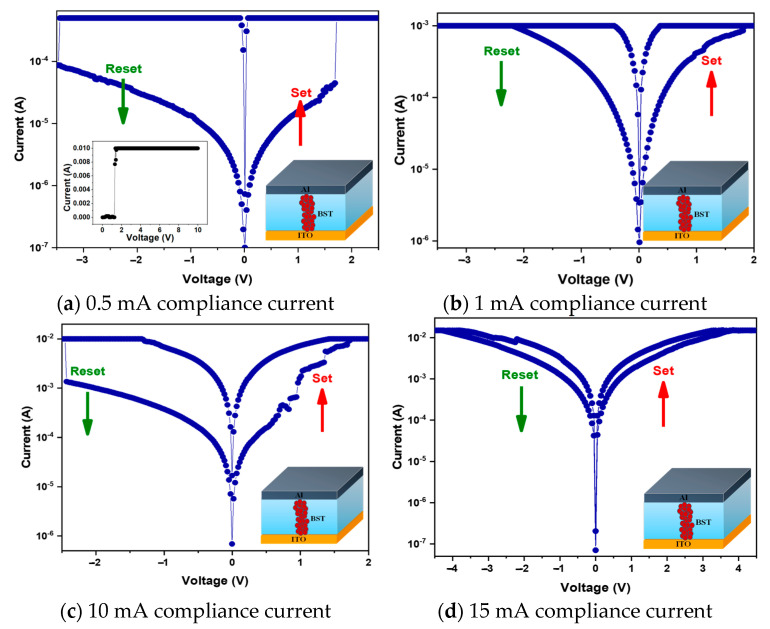
The *I-V* curves of BSTM-film RRAM devices for different compliance currents. (red symbol: oxygen ions).

The memory window of the BSTM resistive memory was determined by scanning back and forth with different compliance currents, as shown in Figure 10. For a compliance current of 0.5 mA, the on/off ratio was approximately 1 and the leakage current was approximately 10^−4^ A. However, the operating voltage was higher because the small compliance current resulted in a higher voltage being required for carrier migration after the filament formation, as shown in Figure 10a. For 1 mA compliance current, the operating voltage dropped significantly and the storage window became smaller. We believe that the insufficient compliance current and the simultaneous decrease in the operating voltage led to a decrease in the switching speed between the break and formation of the resistive filament in the memory element and an unstable state at the set terminal, as shown in Figure 10b. For a compliance current of 10 mA, the on/off ratio was about 5, the memory window was significantly increased compared to other compliance current conditions, the leakage current was about 10^−7^ A, and the operating voltage dropped to −1–0.8 V. At this point, the channel formed by the resistive filament in the device was large enough for carriers to pass through quickly under low-voltage operating conditions, as shown in Figure 10c. But for 15 mA compliance current, the memory window decreased and the operating voltage increased because thicker resistive filaments were formed after a larger or excessive current was applied, resulting in incomplete resistive filament breakage and increased leakage current during reset, and the high current condition would easily cause the film to collapse, as shown in Figure 10d.

**Figure 11:**
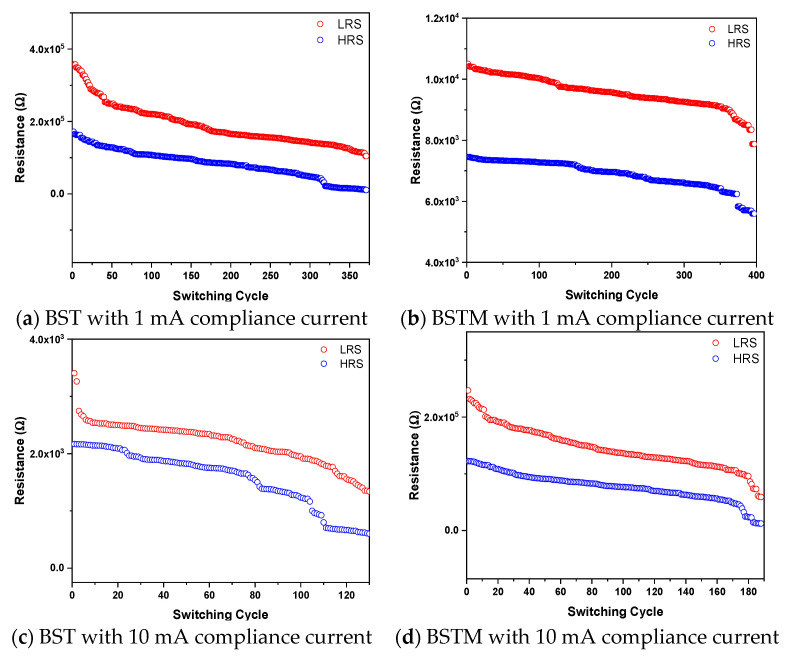
The retention and switching cycle properties of BST- and BSTM-film RRAM devices for different compliance currents.

For 1 mA compliance current, the number of operations of the BST resistive memory was increased to about 370, and the current degradation was slightly improved, as shown in Figure 11a. Meanwhile, the switching between the HRS and LRS of the BSTM resistive memory became more pronounced, and the number of operations was increased to about 400, as shown in Figure 11b. Next, at a limiting current of 10 mA, the difference between the HRS and LRS of the BST resistive memory was more stable, but as the number of operations increased, the current gradually decreased, and the switching characteristic disappeared after about 130 operations, as shown in Figure 11c. The reason for this is thought to be that prolonged operation at high currents causes the film in the device to collapse and carriers to accumulate at the junction, creating a barrier that prevents electrons from passing through efficiently. In Figure 11d, it can be seen that between the HRS and LRS of the BSTM resistive memory, although there is a gap of five orders in the memory window at the beginning, the memory window gradually deteriorates due to the high current. In addition to the switching stability of the BSTM memory, the decrease in current with the number of operations is slightly smaller compared to the BST resistive memory, and the switching characteristic gradually disappears after 190 operations.

**Figure 12:**
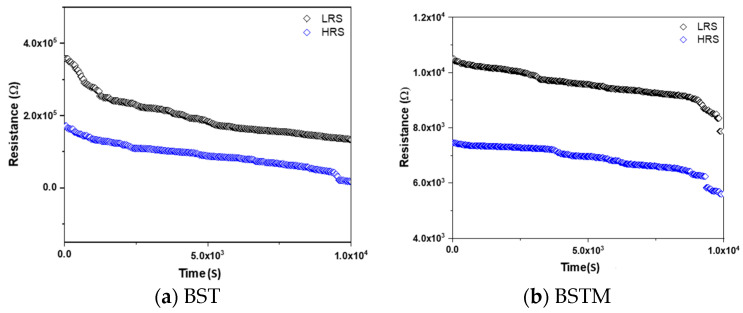
The reliability properties of BST- and BSTM-film RRAM devices for different compliance currents.

In addition to the number of consecutive operations of the device, the retention time is also an important indicator. Therefore, in this section, a 10,000 s test was performed on the BST and BSTM resistive memory devices to determine the on/off retention time of the devices. Figure 12 shows that these devices still maintained a stable HRS and LRS after the 10,000 s test.

**Figure 13:**
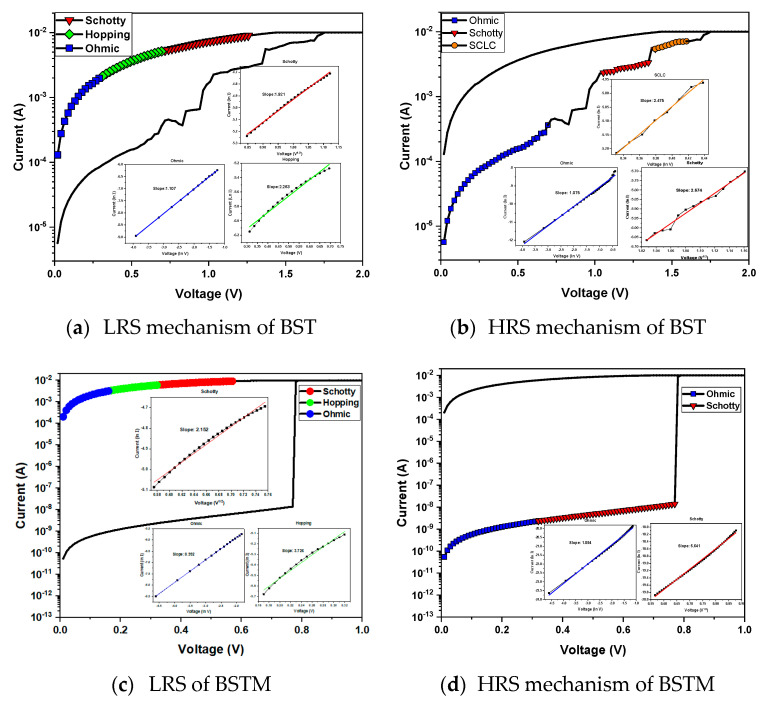
Conduction mechanism analysis of BST- and BSTM-film RRAM devices for different compliance currents.

Figure 13a,b show the fitting results of the BST resistive memory with 10 mA compliance current. The experimental results show that at low voltages, the Ln(I)-Ln(V) slopes of the HRS and LRS fitting results were both 1, consistent with ohmic conduction. At such low voltages, a small number of electrons are conducted through defects in the impedance layer to form a current. When the voltage is increased, the electrons are thermally excited and gain enough energy to jump between the defects. For the LRS, the fitting result of Ln(I)-V was linear, indicating hopping conduction. The higher voltage then causes the electrons to cross the energy barrier, resulting in Schottky emission conduction. Therefore, for the HRS and LRS, the fitting results of Ln(I)-V^1/2^ were linear. In the HRS resistance state, the Ln(I)-Ln(V) slope was 2 at higher voltages, indicating the space charge-limited conduction caused by the rapid passage of electrons at high voltages after they were trapped by the defects in the impedance layer and reached saturation.

The electrical conduction mechanism in the *I-V* curve fitting of the resistive memory devices plays an important role, and the related measurement method is discussed and experimental results are provided in our previous study [12]. The conduction mechanism of BSTM resistive memory was analyzed, as shown in Figure 13c,d, for the experimental results. At low voltages, the HRS and LRS conduction mechanisms both had a fitting slope of 1, thus conforming to ohmic conduction. As the voltage increased, the electrons were thermally excited, causing them to jump between defects on the LRS, resulting in hopping conduction. A further increase in voltage then gave the electrons enough energy to cross the energy barrier, resulting in Schottky emission conduction.

**Figure 14:**
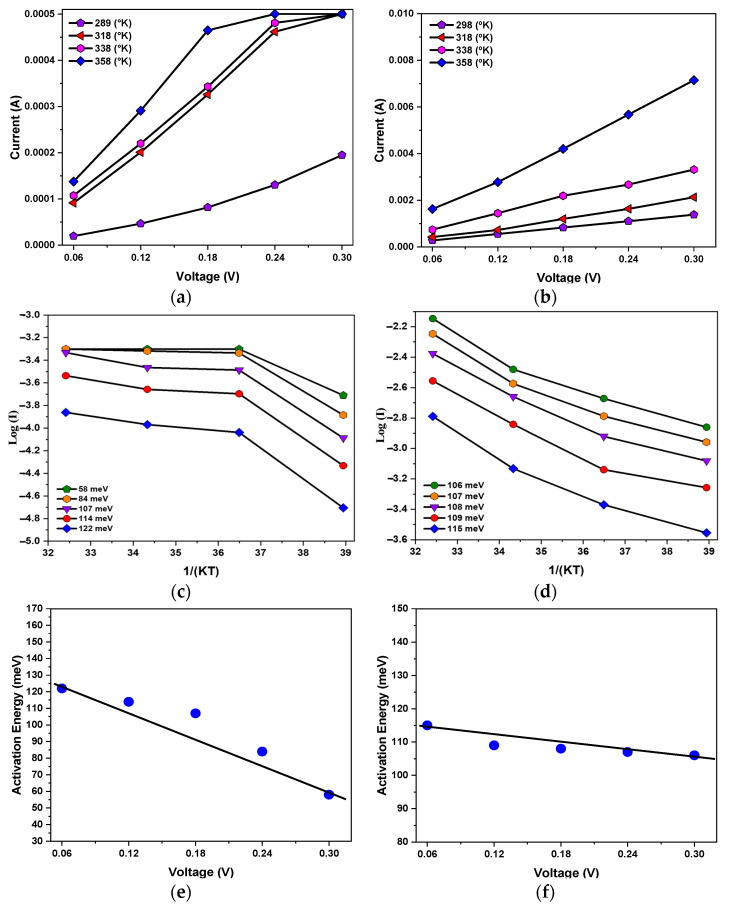
The activation energy versus applied voltage of BST-film RRAM devices for different compliance currents: (**a**) temperature variation with 0.5 mA compliance current; (**b**) temperature variation with 10 mA compliance current; (**c**) electronic activation energy for 0.5 mA compliance current; (**d**) electronic activation energy for 10 mA compliance current; (**e**) voltage and activation energy for 0.5 mA compliance current; (**f**) voltage and activation energy for 10 mA compliance current. (Blue dots: activation energy versus the applied voltage).

Temperature-dependent current measurements were performed on the test films at 289, 318, 338, and 358 °K with a compliance current of 0.5 mA, and the results are shown in Figure 14a. The current increased with increasing temperature, which is consistent with the theory that current is proportional to temperature, as described in the literature [26].

Figure 14c shows the energy required for electron hopping at voltages ranging from 0.06 to 0.3 V. The electron activation energies calculated from the experimental results were 122, 114, 107, 84, and 58 meV. The activation energy was then plotted against the voltage, and the energy of an electron crossing the energy barrier when an external electric field was applied was calculated by the formula to be 138 meV, and the average electron hopping distance was 0.405 μm, as shown in Figure 14e.

**Figure 15:**
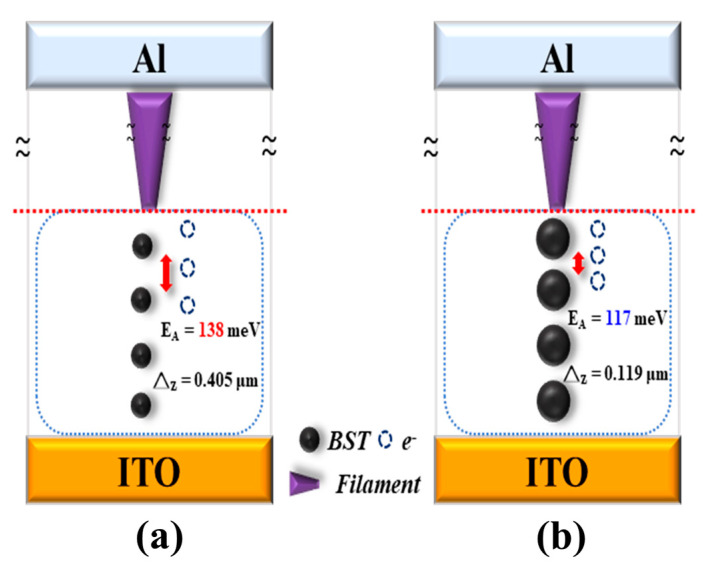
Electron hopping model of BST thin film RRAM devices for the compliance currents of (**a**) 0.5 mA, and (**b**) 10 mA.

Next, at a compliance current of 10 mA, the electron activation energy and the electron hopping distance were derived using the same calculation procedure as for the 0.5 mA compliance current, as shown in Figure 14b. The activation energy calculations were performed with the same voltage of 0.06–0.3 V, and the results were 115, 109, 108, 107, and 106 meV, as shown in Figure 14d. The electron activation energy was then plotted against the voltage to obtain the energy required to cross the energy barrier without an external electric field of 117 meV, which was then used to calculate the average electron hopping distance of 0.119 μm with the formula, as shown in Figure 14f, and an electron hopping distance model was obtained based on the experimental results in assume physical model, as shown in Figure 15. 

**Figure 16:**
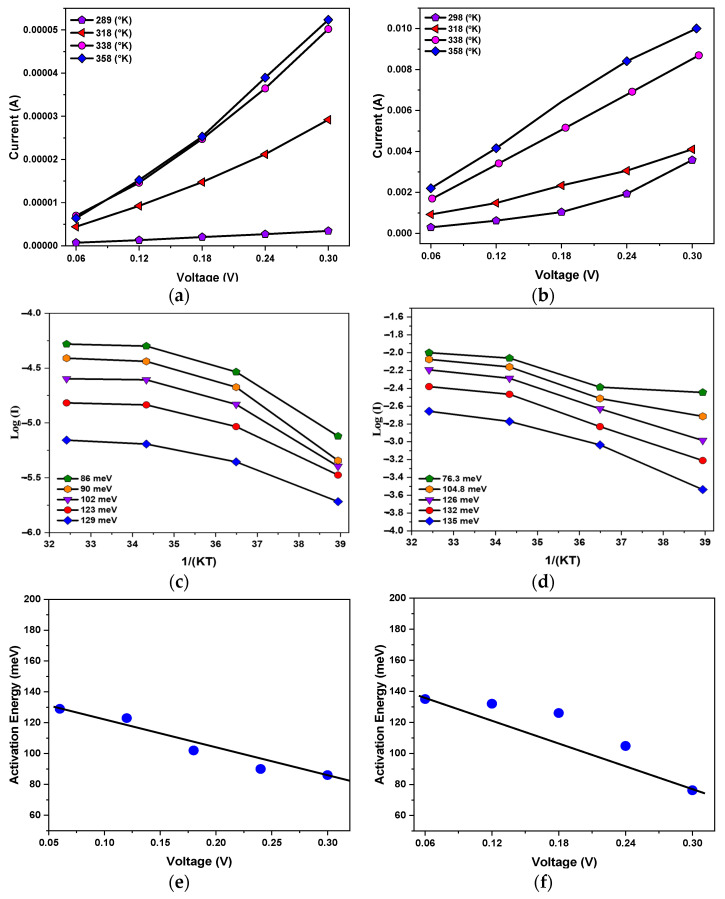
The activation energy versus applied voltage of BSTM-film RRAM devices for different compliance currents: (**a**) temperature variation with 0.5 mA compliance current; (**b**) temperature variation with 10 mA compliance current; (**c**) electronic activation energy for 0.5 mA compliance current; (**d**) electronic activation energy for 10 mA compliance current; (**e**) voltage and activation energy for 0.5 mA compliance current; (**f**) voltage and activation energy for 10 mA compliance current. (Blue dots: activation energy versus the applied voltage).

Temperature-dependent current measurements were performed on the test films at 289, 318, 338, and 358 K with a compliance current of 0.5 mA, and the results are shown in Figure 16a. The current increased with increasing temperature, which is consistent with the theory that current is proportional to temperature, as described in the literature [27].

Figure 16c shows the energy required for electron hopping at voltages ranging from 0.06 to 0.3 V. The electron activation energies calculated from the experimental results were 129, 123, 101.9, 90, and 86 meV. The activation energy was then plotted against the voltage, and the energy of an electron crossing the energy barrier when an external electric field was applied was calculated by the formula to be 140 meV, and the average electron hopping distance was 0.24 μm, as shown in Figure 16e.

**Figure 17:**
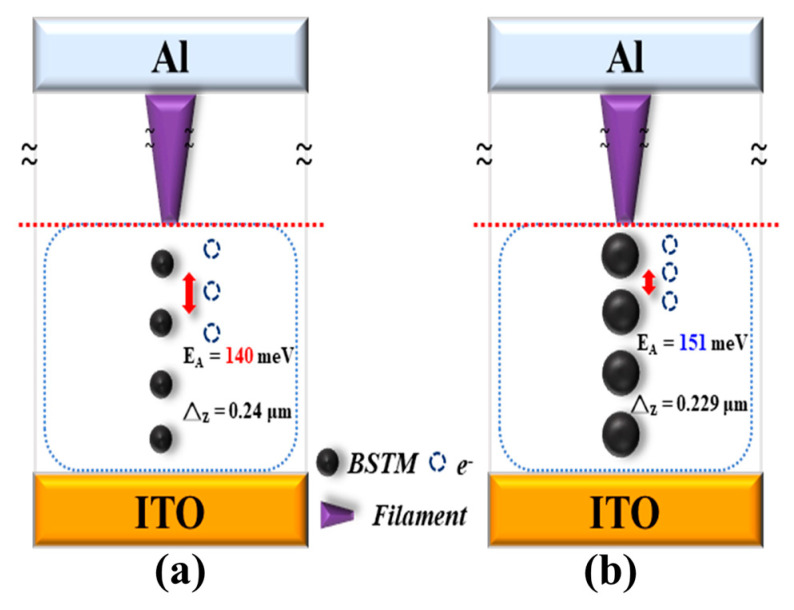
Electron hopping model of BSTM thin film RRAM devices for the compliance currents of (**a**) 0.5 mA, and (**b**) 10 mA.

Next, temperature-dependent current measurements were performed on the BSTM resistive memory at a compliance current of 10 mA. The electron activation energy and the electron hopping distance were derived using the same calculation procedure as for the 0.5 mA compliance current, as shown in Figure 16b. The activation energy calculations were performed with the same voltage of 0.06–0.3 V, and the results were 135, 132, 126, 104.8, and 76 meV, as shown in Figure 16d. The electron activation energy was then plotted against the voltage to obtain the energy required to cross the energy barrier without an external electric field of 151 meV, which was then used to calculate the average electron hopping distance of 0.229 μm with the formula as shown in Figure 16f, and an electron hopping distance model was obtained based on the experimental results as shown in Figure 17.

## 4. Conclusions

Calcite powders were sintered by the one-time sintering method and the solid-state reaction method, and precursor solutions were prepared by a new ball milling technique for deposition of films by the coating method. The addition of Mn can produce a small amount of Mn^4+^ to replace Ti^4+^ in Ba_0.6_Sr_0.4_Ti_1-x_Mn_x_O_3_, which will gradually increase its crystallinity. Therefore, the chemical formula Ba_0.6_Sr_0.4_Ti_0.9_Mn_0.1_O_3_ is considered to be the best result. The BSTM resistive memory had an on/off ratio of about 5, a leakage current of about 10^−7^ A, and its operating voltage dropped to 1 V under the same measurement conditions. As a result, BSTM resistive memories have low power dissipation and can be operated at low voltages with a good memory window. Finally, the hopping distance was larger in the BSTM resistive memory at a compliance current of 10 mA, while it was smaller in the BSTM resistive memory at a compliance current of 0.5 mA. Therefore, it is hypothesized that the resistivity of the BSTM resistive memory is larger at lower currents, and many atoms accumulate at the interface between the impedance layer and the metal to form an energy barrier, leading to a significant increase in the hopping distance.

## Figures and Tables

**Table 1:** XRD results of the BST material for one-time sintering method and solid-state reaction method.

	Solid-State Reaction Method	One-Time Sintering Method
WHM (101)	0.385	0.245
FWHM (111)	0.342	0.235
Theoretical density (g/cm^3^)	5.566	5.563
Average grain diameter (μm)	0.174	0.268
Crystallinity (%)	96.123	97.223

**Table 2:** Analysis of AFM of BST materials for different sintering methods.

(Unit: nm)	Solid-State Reaction Method	One-Time Sintering Method
Average roughness R_a_	72.1	125
Root mean square roughness R_q_	93.7	159
Maximum height roughness R_max_	909	1264

**Table 3:** XRD analysis for different Mn proportions of BSTM materials.

(Unit: nm)	x = 0.05	x = 0.1	x = 0.15	x = 0.2
FWHM (110)	0.279	0.234	0.308	0.735
FWHM (111)	0.246	0.227	0.259	0.205
Theoretical density (g/cm^3^)	5.56	5.5	5.486	5.475
Average grain diameter (μm)	0.242	0.244	0.221	0.379
Crystallinity (%)	98.523	98.577	98.276	95.653

**Table 4:** AFM analysis for different Mn proportions of BSTM materials.

(Unit: nm)	x = 0.05	x = 0.1	x = 0.15	x = 0.2
Average roughness R_a_	100	86.5	102	118
Root mean square roughness R_q_	128	111	128	151
Maximum height roughness R_max_	1041	1044	1070	1283

## Data Availability

The original contributions presented in the study are included in the article, further inquiries can be directed to the corresponding author/s.
